# 3-Deazaadenosine keeps senescence at bay

**DOI:** 10.18632/aging.204625

**Published:** 2023-03-24

**Authors:** Ana Guerrero, Jesús Gil

**Affiliations:** 1 MRC London Institute of Medical Sciences (LMS), London, UK; 2 Institute of Clinical Sciences (ICS), Faculty of Medicine, Imperial College London, UK

**Keywords:** 3-deazaadenosine, senescence, aging, SAM

Cellular senescence is a key mechanism to prevent the expansion of old, damaged or cancerous cells. Senescent cells undergo an irreversible cell growth arrest and secrete immunomodulatory cytokines that activate immune surveillance [1]. In that way, senescence helps preserve tissue homeostasis and acts as a natural barrier against tumorigenesis. Paradoxically, the aberrant accumulation of senescent cells, observed in aging and age-related diseases, comes together with multiple negative consequences. The discovery that eliminating these lingering senescent cells improves many age-related phenotypes [2], has led the aging field into a relentless search for strategies to therapeutically target senescence. Senolytic drugs, i.e., drugs that selectively kill senescent cells, show promising results in a myriad of preclinical models and are starting to move into clinical trials.

We previously established a high-throughput screening platform that resulted in the identification of the senolytic activity of cardiac glycosides such as ouabain and digoxin [3]. While senolytic drugs hold huge promise, recent work has shown that eliminating certain senescent cells such as liver sinusoidal endothelial cells (LSECs) can be detrimental [4]. Therefore, it is important to explore alternative approaches to mitigate the negative effects of senescence. With that in mind, we adapted that pipeline to search for drugs able to alleviate senescence, successfully identifying 3-deazaadenosine (3DA) [5].

We screened more than 1,500 small molecules in cultures of human primary foetal lung fibroblasts and shortlisted those drugs alleviating both oncogene-induced senescence and senescence caused by telomere uncapping. Among the candidates, we decided to explore further the activities of 3DA, an inhibitor of the S-Adenosyl-L-Homocysteine Hydrolase (AHCY) enzyme. AHCY catalyses the hydrolysis of S-adenosylhomocysteine (SAH), a by-product of SAM-dependent methyltransferase reactions, to homocysteine and adenosine. Importantly, SAH acts as an inhibitor of methyltransferases.

When senescence was triggered in the presence of 3DA, the associated cell growth arrest was delayed, and 3DA-treated cells also showed lower levels of p16^INK4a^ expression and senescence-associated-β-galactosidase activity (SA-β-Gal) than vehicle treated cells. Of note, these effects were due to on-target activities of 3DA, as similar results were obtained by knocking down AHCY using two different short hairpins RNAs. As a consequence of inhibiting AHCY is the disruption of SAM-dependent methyltransferase reactions, we investigated whether methylation of specific histone residues were affected on 3DA-treated senescent cells. We found a global reduction in the levels of H3K36me3, an epigenetic modification often associated with actively transcribed gene bodies. To get further insights into the role of 3DA in senescence, we combined transcriptomic analysis with genomic mapping of H3K36me3 by ChIP-seq. The results showed that 3DA, through its effect on H3K36me3 levels, influences key elements of the senescence transcriptional program including components of the senescence-associated secretory phenotype (SASP), one of the hallmarks of senescence and accountable for its detrimental pro-inflammatory activities.

In addition to fuelling inflammation, senescence favours aging by directly impacting the regenerative potential of stem cell populations [6]. Treatment with 3DA decreases the incidence of senescence among skeletal muscle stem cells from very old mice. When these geriatric 3DA-treated cells were transplanted into pre-injured muscles of immunodeficient recipient mice, they showed greater engraftment and regenerative capacities than when mice were engrafted with geriatric vehicle-treated cells. These results highlight how 3DA and similar therapies aimed at alleviating senescence could boost tissue repair and regeneration, mitigating the effects of age-related deterioration. Moreover, we demonstrated that 3DA treatment helps to expand human umbilical cord blood cells *ex vivo* for transplant purposes, as low numbers and low self-renewal capacity are the current main barriers preventing their widespread use in the clinic.

A recent preprint [7] suggests that low intracellular SAM levels during senescence prevent reprogramming. Although not formally tested, our results imply that 3DA indirectly lowers SAM levels because of the accumulation of SAH, leading to a delay in senescence that favourably impacts on stem cells and tissue homeostasis. A potential explanation for this discrepancy is that high levels of SAM may be required to support acute epigenetic remodelling during senescence initiation but may not be necessary and hence decline once senescence has been established. Importantly, our study focused on short term effects of *in vitro* and *ex vivo* 3DA treatments in senescence as opposed to long-term effects. In any case, the new study alongside ours bring to light the relevance of the regulation of metabolites such as SAM and SAH, and the changes in histone methylation patterns for senescence fate and reprogramming. This will spearhead new avenues of research due to its therapeutic potential.

Overall, we envision strategies preventing or delaying senescence as a complementary or alternative option to senolytics in certain scenarios. Particularly, acute, time-limited, *ex vivo* treatment of cells that are to be transplanted might limit senescence and help with engraftment while limiting the detrimental effects associated with inhibiting senescence *in vivo*. Since inhibiting senescence could negatively impact wound-healing [8] or increase the risk of tumours, any such therapies have to be carefully considered. To shed light on this matter, future studies should investigate long-term outcomes of 3DA treatment and its potential effects on epigenetic marks, including on DNA methylation. For clinical applications, an effective therapeutic window has yet to be determined, carefully assessing potential adverse on-target and off-target effects.
